# Smartwatch-based pelvic tracking system for augmented reality navigation in total hip arthroplasty: a preliminary clinical study

**DOI:** 10.1186/s42836-026-00441-3

**Published:** 2026-11-02

**Authors:** Ryohei Takada, Ryo Inokuchi, Yusuke Amano, Riko Yamashita, Toshitaka Yoshii, Hideyuki Koga

**Affiliations:** Department of Orthopaedic Surgery, Institute of Science Tokyo Hospital, 1-5-45 Yushima, Bunkyo-Ku, Tokyo, 113-8519 Japan

**Keywords:** Smartwatch, Augmented reality, Pelvic tracking, Navigation system, Total hip arthroplasty, Cup alignment

## Abstract

**Background:**

Accurate assessment of pelvic orientation is essential for acetabular cup placement during total hip arthroplasty (THA). Conventional pelvic tracking systems generally rely on dedicated tracking hardware and optical tracking infrastructure. We developed a smartwatch-based pelvic tracking system integrated with an augmented reality (AR) navigation platform and evaluated its preliminary clinical performance.

**Methods:**

This retrospective study included 30 hips of 27 consecutive patients who underwent primary THA using a smartwatch-based pelvic tracking navigation system between May 2025 and April 2026. The system utilizes a commercially available smartwatch fixed to the iliac crest as a pelvic tracking device and communicates orientation information to a smartphone-based AR navigation platform via Bluetooth. Acetabular cup orientation was evaluated using postoperative CT. Absolute radiographic inclination (RI) and anteversion (RA) errors were calculated as absolute differences between the final intraoperative navigation measurements and postoperative CT measurements.

**Results:**

The mean absolute RI error was 2.8° ± 2.1°, and the mean absolute RA error was 3.6° ± 2.2°. Postoperative CT measurements were within 5° of the final intraoperative navigation measurements for RI and RA in 86.7% and 70.0% of hips, respectively. Navigation was successfully completed in all 30 hips, with no conversion to a backup technique. Additional bench testing demonstrated reproducible smartwatch reattachment and reduction of time-dependent relative orientation differences following FPP invocation.

**Conclusions:**

This study demonstrated the feasibility of integrating a commercially available smartwatch into a pelvic tracking system for THA navigation. The smartwatch-based pelvic tracking platform achieved a preliminary range of cup-positioning accuracy without requiring continuous optical visualization of a pelvic reference marker while utilizing a compact and widely available consumer wearable device. This study represents the first step toward wearable-device-based navigation in arthroplasty.

**Supplementary Information:**

The online version contains supplementary material available at https://doi.org/10.1186/s42836-026-00441-3.

## Background

Accurate acetabular cup positioning is essential for successful total hip arthroplasty (THA). Malpositioning of the acetabular component is associated with postoperative dislocation, impingement, accelerated wear, reduced range of motion, and patient dissatisfaction [1, 2]. To improve the accuracy of cup placement, various computer-assisted technologies have been developed, including computed tomography (CT)-based navigation systems, imageless navigation systems, robotic-assisted systems, and augmented reality (AR)-based navigation systems [3–5].

An accurate assessment of pelvic orientation is fundamental to these navigation systems because changes in pelvic position during surgery may influence cup alignment. Therefore, many navigation systems utilize pelvic tracking devices to continuously monitor pelvic orientation throughout the procedure [6, 7]. However, conventional pelvic tracking systems generally rely on dedicated tracking hardware and optical tracking infrastructure, which may increase system complexity, equipment size, and cost.

Recent advances in AR technology and consumer electronics have facilitated the development of portable navigation systems for THA. Smartphone-based AR navigation systems have demonstrated satisfactory accuracy with reduced hardware requirements and improved portability [8–10]. However, in conventional marker-based AR navigation systems, the pelvic reference marker must remain within the field of view of the camera to continuously update the pelvic orientation. Consequently, the real-time display of radiographic inclination (RI) and radiographic anteversion (RA) relative to the current pelvic position generally requires continuous recognition of the pelvic marker during cup implantation.

Commercially available smartwatches are unique wearable platforms equipped with inertial sensors, wireless communication capabilities, and compact hardware. Previous studies have demonstrated that inertial sensors embedded in consumer smartwatches provide reliable motion-tracking performance with angular velocity measurements comparable to those of research-grade IMUs, supporting their potential use in clinical motion-tracking applications [11]. Continuous transmission of orientation information via Bluetooth allows real-time tracking of device orientation without continuous optical visualization. Therefore, we integrated a smartwatch-based pelvic tracking system with an AR navigation platform for THA. This study aimed to evaluate the preliminary clinical feasibility and cup-positioning accuracy of this smartwatch-based pelvic tracking system in patients undergoing THA.

## Methods

### Study design and patients

This study was conducted at a single university hospital in accordance with the principles of the Declaration of Helsinki. This study protocol was approved by the institutional review board, and informed consent was obtained from all patients before surgery. This retrospective study included 30 hips of 27 consecutive patients who underwent primary THA using a smartwatch-based pelvic tracking navigation system between May 2025 and April 2026 at our institution. Three patients underwent bilateral procedures during the study period. In patients undergoing bilateral THA, smartwatch fixation and pelvic registration were performed independently for each hip because the smartwatch was removed and refixed for the contralateral procedure as part of the sterile surgical workflow.

All patients were diagnosed with hip osteoarthritis. The study cohort consisted of three men and 24 women with a mean age of 68.8 (range: 53–84) years. All procedures were performed by three experienced arthroplasty surgeons using the supine anterolateral approach.

### Smartwatch-based pelvic tracking system and surgical procedure

#### System overview

A smartwatch-based pelvic tracking system developed by Xelha Medical Co., Ltd. (Nagano, Japan) was used in this study. The system consisted of an iPhone 16e (Apple Inc., Cupertino, CA, USA) and an Apple Watch Series 10 (Apple Inc., Cupertino, CA, USA), which communicated wirelessly via Bluetooth. The smartwatch was rigidly fixed to the iliac crest using two percutaneous pins and functioned as a pelvic tracking device by continuously transmitting orientation information derived from its inertial sensors, including the gyroscope and accelerometer, to the smartphone. Changes in smartwatch orientation accompanying pelvic rotation were continuously transmitted to the smartphone and used to update the pelvic orientation reference for real-time calculation of RI and RA. The smartphone served as the navigation platform, generating and reconstructing the functional pelvic plane (FPP), and calculating the RI and RA in real time.

#### Pelvic registration

**Fig. 1:**
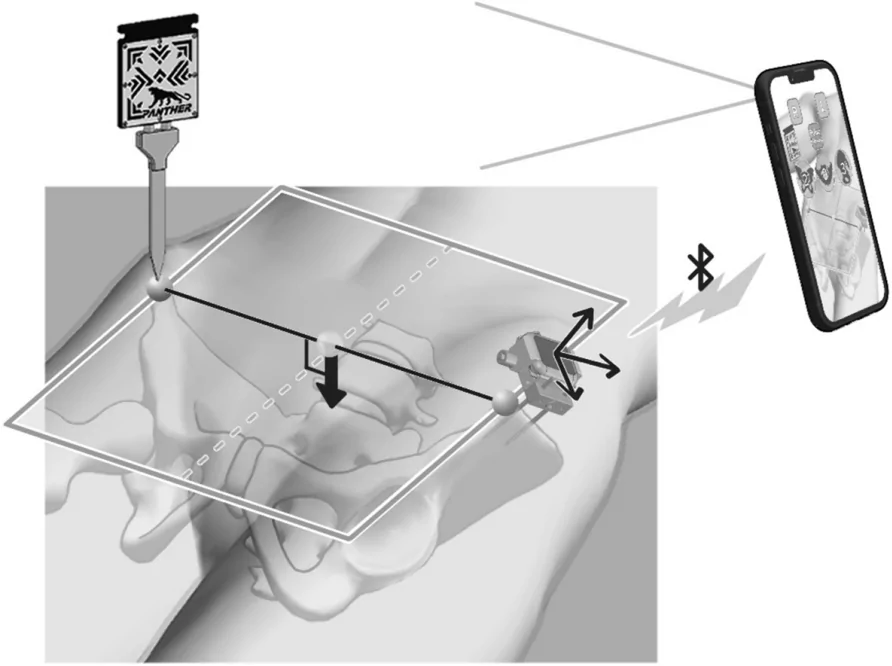
FPP registration and establishment of the spatial relationship between the FPP and smartwatch orientation. During FPP registration, the smartphone generated a functional pelvic plane (FPP) using AR-based registration of the bilateral anterior superior iliac spines (ASISs), together with gravity information. Simultaneously, the orientation information from the smartwatch fixed to the pelvis was transmitted to the smartphone via Bluetooth, and the spatial relationship between the registered FPP and smartwatch orientation was stored. The two spheres represent the bilateral ASISs and the rectangular plane represents the FPP

Before surgery, the smartwatch was rigidly fixed to the iliac crest using two percutaneous pins. While the pelvis remained in its initial position, pelvic registration was performed by generating the FPP on the smartphone using AR-based registration of the bilateral anterior superior iliac spines (ASISs) together with gravity information obtained from the device sensors [10]. Simultaneously, the current orientation of the smartwatch was transmitted to the smartphone via Bluetooth, and the smartphone calculated and stored the spatial relationship between the registered FPP and the smartwatch orientation (Fig. 1).

Following pelvic registration, the smartwatch was temporarily detached during surgical exposure and reattached using a dedicated fixation mechanism before cup implantation. A dedicated fixation mechanism enabled reproducible reattachment of the smartwatch in the same predefined position and orientation relative to the pelvis. The FPP invocation procedure is described below.

#### FPP invocation

Immediately before cup implantation, an FPP invocation procedure was performed. During this procedure, the smartphone recognized an AR marker displayed on the smartwatch screen while simultaneously receiving the current orientation of the smartwatch via Bluetooth. Using the previously stored spatial relationship between the registered FPP and smartwatch orientation, together with the current smartwatch orientation, the smartphone reconstructed the current FPP.

The FPP invocation procedure was introduced to compensate for the potential relative drift between the smartphone and smartwatch that may occur over time. Because the smartphone and smartwatch independently estimate their orientations, a time-dependent relative orientation error may accumulate between the two devices. The spatial relationship between the registered FPP and smartwatch orientation established during pelvic registration allowed reconstruction of the current FPP without repeating ASIS registration. Following FPP invocation, the reconstructed FPP was used as the reference plane for real-time calculation and display of RI and RA during cup implantation. During clinical use, the system required FPP invocation when 10 min had elapsed since the previous invocation before navigation could be continued, thereby ensuring periodic re-establishment of the relative orientation relationship between the smartwatch and smartphone.

#### Cup implantation

**Fig. 2:**
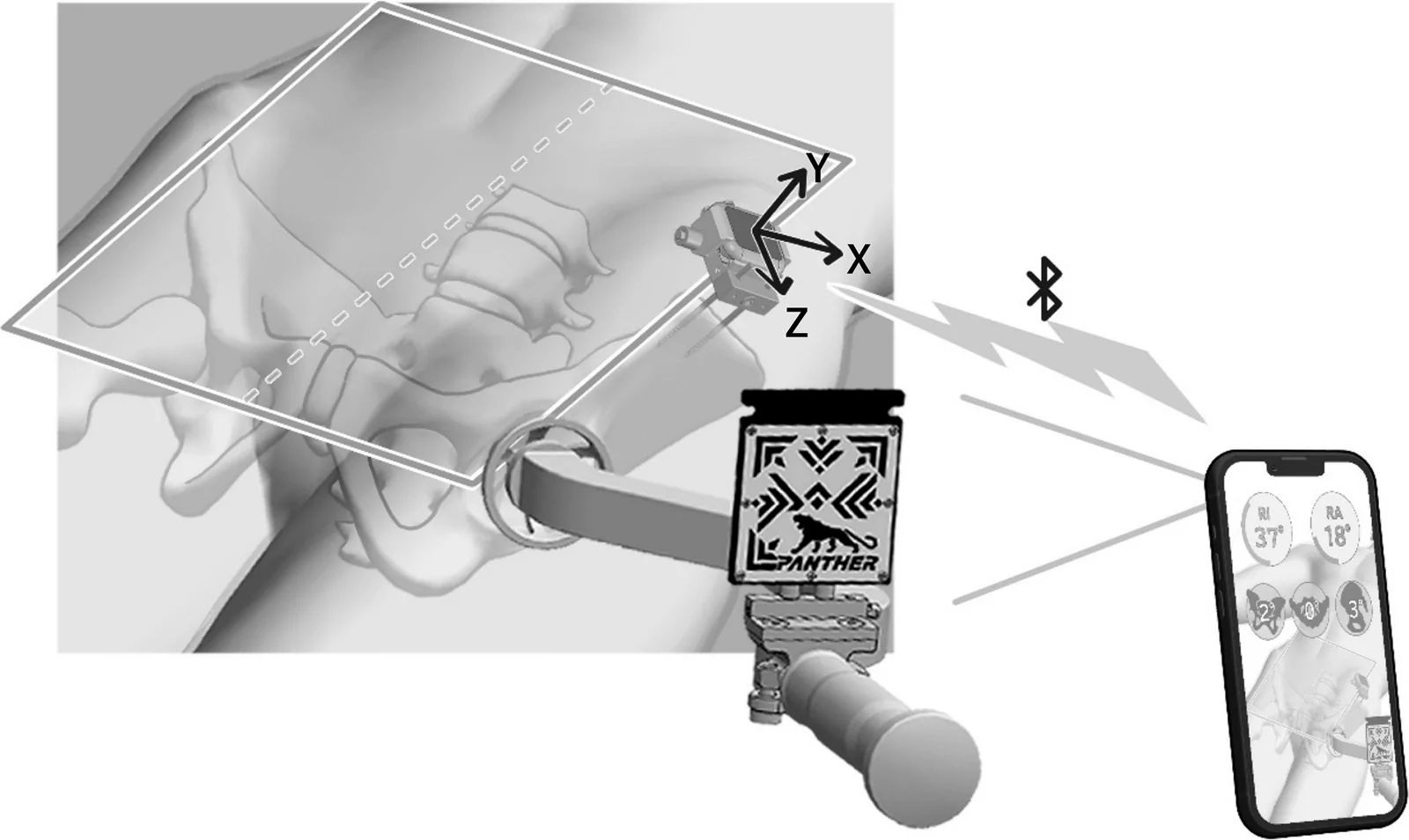
Reconstruction of the current FPP and calculation of cup orientation during cup implantation. During cup implantation, the current orientation of the smartwatch was transmitted to the smartphone via Bluetooth. Using the previously stored spatial relationship established during the FPP registration, the smartphone reconstructs the current FPP corresponding to the current pelvic orientation**.** By recognizing the AR marker attached to the cup impactor, the system calculates and displays the RI and RA in real-time relative to the reconstructed FPP

**Fig. 3:**
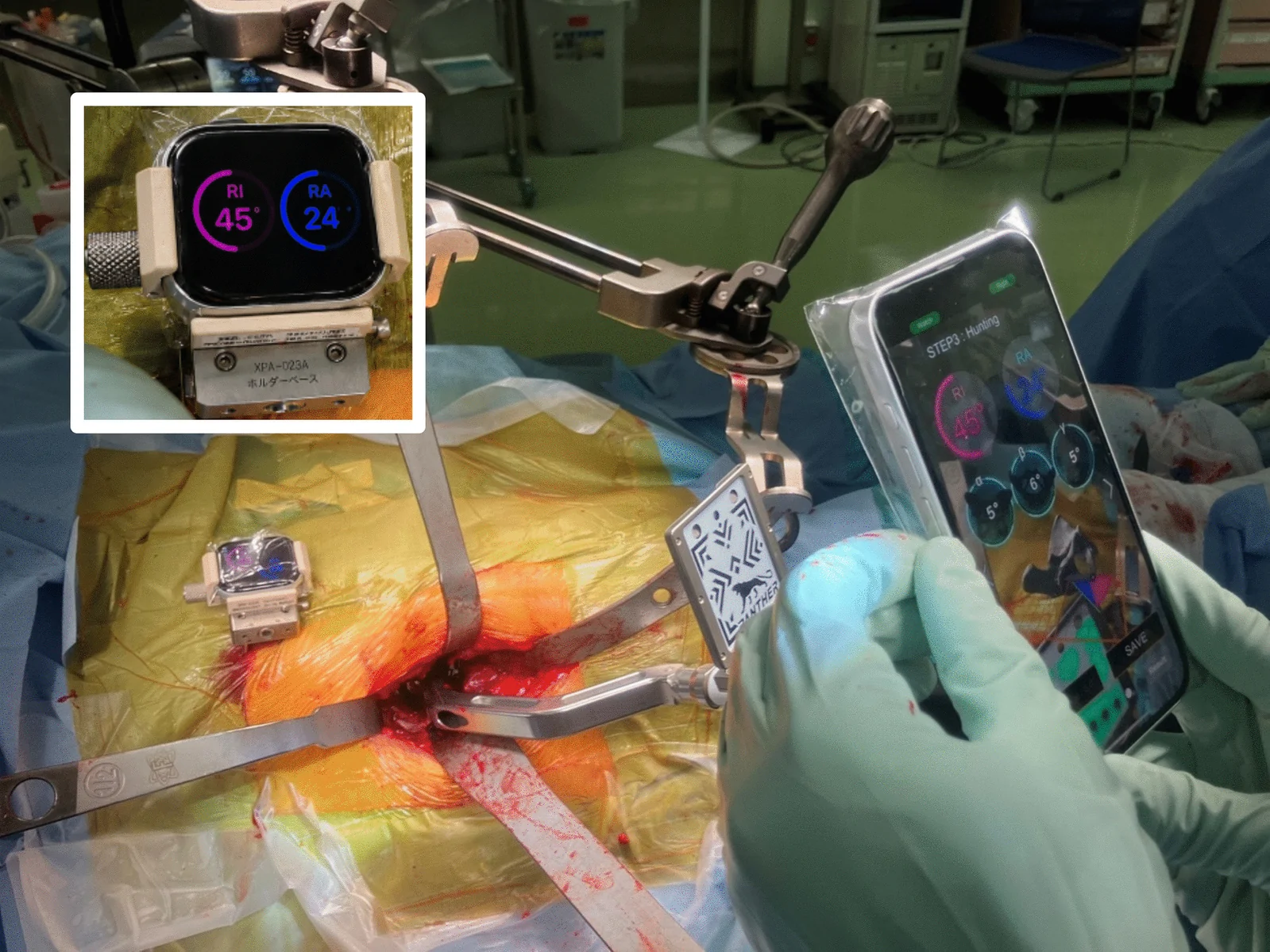
Intraoperative use of the smartwatch-based pelvic tracking navigation system during THA. The smartwatch fixed to the pelvis and smartphone simultaneously displayed the RI and RA during cup implantation. An enlarged view of the smartwatch display is presented in the inset. The AR marker attached to the cup impactor was recognized by the smartphone camera and is highlighted in green, enabling the real-time calculation of cup orientation relative to the reconstructed FPP

After reconstruction of the current FPP, the smartphone recognized the AR marker attached to the cup impactor and calculated the RI and RA relative to the reconstructed FPP in real time (Fig. 2). Unlike conventional marker-based AR navigation systems, continuous visualization of a pelvic reference marker was not required during cup implantation because the current FPP was reconstructed using the current smartwatch orientation together with the previously stored spatial relationship to the registered FPP. The calculated RI and RA were displayed simultaneously on both the smartphone and smartwatch, allowing the surgeon to continuously monitor cup orientation throughout implantation without continuous visualization of the pelvic reference marker (Fig. 3). The intended cup orientation was generally an RI of 40° and an RA of 15°–25°, with the final target orientation determined intraoperatively by the operating surgeon taking the combined anteversion concept into consideration. The final RI and RA values displayed by the navigation system at the completion of cup implantation were recorded as the final intraoperative navigation measurements.

### Outcome assessment

The primary outcome measures were the absolute errors of RI and RA. Postoperative CT scans were obtained for all patients, and cup orientation was measured using the ZedHip software (Lexi Co., Ltd., Tokyo, Japan) by a single evaluator (RI). The RI and RA were defined according to the classification described by Murray et al. [12] and measured relative to the FPP coordinate system.

The absolute RI and absolute RA errors were defined as the absolute differences between the final intraoperative navigation measurements and the corresponding postoperative CT measurements, respectively.

$$\mathbf{Absolute}\;\mathbf{RI}\;\mathbf{error}=\mathbf{\left|{\mathbf{Final}\;\mathbf{intraoperative}\;\mathbf{navigation}\;\mathbf{RI}-\mathbf{Postoperative}\;\mathbf{RI}}\right|}$$

$$\mathbf{Absolute\;RA\;error}=\left|\mathbf{Final\;intraoperative\;navigation\;RA}-\mathbf{Postoperative\;RA}\right|$$

Signed differences were also calculated as the postoperative CT measurement minus the final intraoperative navigation measurement for both RI and RA.

To assess intra-observer reproducibility of postoperative CT measurements, all 30 hips were remeasured by the same evaluator in a blinded manner.

In addition to cup positioning accuracy, postoperative complications occurring within 3 months of surgery, including dislocation, periprosthetic fracture, and surgical site infection, were recorded. Technical issues related to the navigation procedure, including registration failure requiring repeat registration, unsuccessful FPP invocation, Bluetooth disconnection, clinically relevant data-transmission delay or display interruption, and conversion to a backup navigation technique, were also recorded.

### Technical validation

Additional bench testing was performed to evaluate the reproducibility of the smartwatch fixation mechanism and time-dependent relative orientation drift between the smartwatch and smartphone. First, detach–reattach reproducibility was assessed by repeatedly detaching and reattaching the smartwatch 20 times while the pelvic model and other components of the experimental setup remained fixed. Changes in three-dimensional orientation and navigation-derived RI and RA were recorded relative to the initial reference orientation.

Second, time-dependent relative orientation drift and the effect of FPP invocation were evaluated under static conditions. Measurements were obtained at 10, 30, and 60 min, with three independent trials performed at each time point. Orientation differences relative to the initial reference were recorded immediately before and after FPP invocation. Detailed experimental procedures and results are provided in the Supplementary Methods, Supplementary Tables S1 and S2, and Supplementary Figures S1 and S2.

### Statistical analysis

Continuous variables were presented as means ± standard deviations (SDs) with 95% confidence intervals (CIs), where appropriate. Categorical variables were presented as counts and percentages. Absolute RI and RA errors and signed differences between the final intraoperative navigation measurements and postoperative CT measurements were summarized descriptively. Intra-observer reproducibility of postoperative CT measurements was assessed using intraclass correlation coefficients (ICCs) based on a two-way mixed-effects model, absolute agreement, and single measurements, with 95% CIs. Because three patients underwent bilateral THA, a sensitivity analysis was performed using one hip per patient to assess the potential influence of within-patient clustering. For patients who underwent bilateral procedures, the first-operated hip was included in the sensitivity analysis. No formal sample-size calculation was performed; the sample consisted of 30 consecutive hips treated during the study period and was considered an exploratory sample for this preliminary clinical study.

## Results

### Patient characteristics

Thirty hips from 27 patients were included in the analysis. The patient demographics are summarized in Table 1.

**Table 1:** Patient demographics

**Characteristic**	**Value**
Patients, n	27
Hips, n	30
Age, years	68.8 ± 8.2 (53–84)
Sex (male/female)	3/24
Diagnosis	Osteoarthritis (30 hips)

### Clinical performance

The mean absolute RI error was 2.8° ± 2.1° (95% CI, 2.0°–3.6°), whereas the mean absolute RA error was 3.6° ± 2.2° (95% CI, 2.8°–4.4°). The mean signed difference, defined as the postoperative CT measurement minus the final intraoperative navigation measurement, was + 1.05° ± 3.42° (95% CI, − 0.22° to 2.33°) for RI and − 1.75° ± 3.87° (95% CI, − 3.19° to − 0.30°) for RA.

**Fig. 4:**
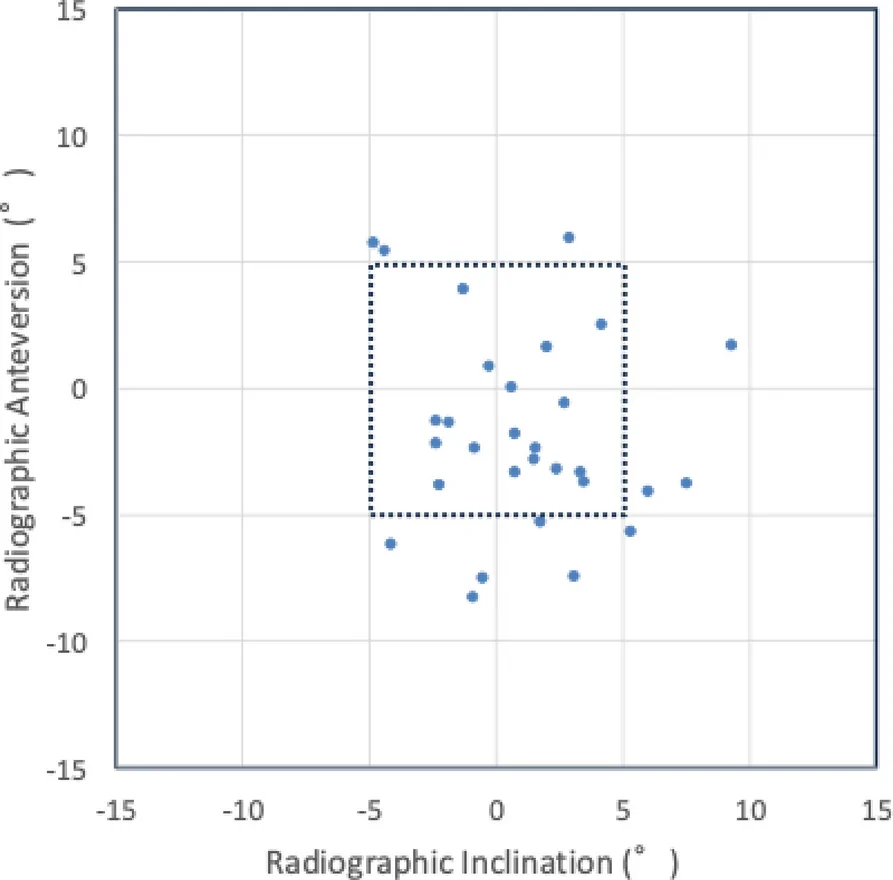
Scatter plot of postoperative RI and RA errors relative to the final intraoperative navigation measurements. Each point represents a hip. The dotted square represents errors within ± 5° of the final intraoperative navigation measurements

Postoperative CT measurements were within 5° of the final intraoperative navigation measurements for RI and RA in 86.7% and 70.0% of hips, respectively (Fig. 4).

Intra-observer reproducibility of the postoperative CT measurements was excellent, with ICCs of 0.976 (95% CI, 0.947–0.989) for RI and 0.976 (95% CI, 0.951–0.989) for RA.

Navigation was successfully completed in all 30 hips without conversion to a backup technique. No registration failure requiring repeat registration, unsuccessful FPP invocation, Bluetooth disconnection, clinically relevant data-transmission delay, or display interruption was observed. No navigation-related intraoperative complications were observed. Furthermore, no postoperative dislocations, periprosthetic fractures, or surgical site infections occurred within 3 months after surgery.

### Technical validation

In the detach–reattach reproducibility test, repeated detachment and reattachment of the smartwatch resulted in small changes in navigation-derived cup orientation. The mean differences from the initial reference were − 0.86° ± 0.40° for RI and + 0.10° ± 0.28° for RA, with maximum absolute differences of 1.4° and 0.9°, respectively.

In the time-dependent drift test, the mean RI differences from the initial reference before FPP invocation were 1.23° ± 0.71°, 2.03° ± 1.00°, and 2.27° ± 0.96° at 10, 30, and 60 min, respectively; after FPP invocation, the corresponding differences were − 0.40° ± 0.70°, 0.80° ± 0.60°, and 0.63° ± 0.86°. The corresponding RA differences before FPP invocation were − 0.03° ± 0.06°, 0.00° ± 0.53°, and 0.77° ± 0.78°, and those after FPP invocation were 0.10° ± 0.10°, 0.27° ± 0.64°, and − 0.13° ± 0.65°, respectively. Detailed results of the technical validation are provided in Supplementary Tables S1 and S2.

### Sensitivity analysis

In the sensitivity analysis restricted to one hip per patient (27 hips from 27 patients), the mean absolute differences were 2.62° ± 1.91° for RI and 3.65° ± 2.27° for RA. The corresponding signed differences were + 0.65° ± 3.22° for RI and − 1.59° ± 4.04° for RA. These results were similar to those of the primary analysis including all 30 hips.

## Discussion

The most important finding of this study is the successful clinical application of a smartwatch-based pelvic tracking system integrated with an AR navigation platform for THA. To our knowledge, this is the first report to describe the use of a commercially available smartwatch as a pelvic tracking device during THA. The system achieved a mean absolute error of 2.8° for RI and 3.6° for RA. Additional bench testing demonstrated reproducible smartwatch reattachment and reduction of time-dependent relative orientation differences following FPP invocation. These findings demonstrate the preliminary clinical feasibility of smartwatch-based pelvic tracking without requiring continuous optical visualization of a pelvic reference marker.

Accurate assessment of pelvic orientation is essential for cup positioning during THA [6]. Conventional navigation systems generally rely on dedicated pelvic tracking hardware and optical tracking infrastructure specifically designed for navigation purposes [3, 4]. Although these systems provide reliable tracking performance, they often require specialized equipment that may increase system complexity and limit portability. In contrast, the present system utilizes a commercially available smartwatch as a pelvic tracking device. Smartwatches are lightweight, compact, and widely distributed worldwide, making them attractive platforms for surgical navigation. The present findings support the feasibility of incorporating a consumer wearable device into a pelvic tracking architecture for THA navigation.

Recent advances in consumer electronics have facilitated the development of portable navigation systems. Smartphone-based AR navigation systems have been introduced as compact and cost-effective alternatives to conventional navigation technologies [8–10]. Building upon these developments, the present system extends the concept of consumer-electronics-based navigation by integrating a smartwatch into the navigation workflow. Unlike conventional marker-based AR navigation systems, which generally require continuous visualization of a pelvic reference marker, the present system allows pelvic orientation to be updated without continuous visualization of a pelvic marker during cup implantation. This feature provides greater flexibility in smartphone positioning, reduces the need for continuous camera alignment with the pelvis, and improves usability during surgery.

The present study demonstrated mean absolute differences of 2.8° for RI and 3.6° for RA between the final intraoperative navigation measurements and postoperative CT measurements. The corresponding signed differences were + 1.05° for RI and − 1.75° for RA. Because this study did not include a direct comparison with other navigation systems or freehand cup placement, these findings should not be interpreted as demonstrating equivalence or superiority to existing techniques. Rather, the principal contribution of this study is the demonstration of a novel smartwatch-based pelvic tracking architecture that enables intraoperative cup-orientation measurement without continuous optical visualization of a pelvic reference marker.

The additional bench testing provided technical support for two key aspects of the proposed tracking architecture. Repeated detachment and reattachment of the smartwatch resulted in mean RI and RA differences of − 0.86° and + 0.10°, respectively, indicating reproducible restoration of the navigation-derived orientation under controlled conditions. In addition, time-dependent relative orientation differences were observed between the smartwatch and smartphone, whereas FPP invocation reduced these differences toward the initial reference orientation. These findings support the use of periodic FPP invocation as a practical mechanism for limiting the influence of relative orientation drift during navigation.

Another notable aspect of the proposed system is its potential for future development. As commercially available wearable devices continue to evolve rapidly, future systems may benefit from improvements in sensor performance, wireless communication, computational power, and device miniaturization. Furthermore, wearable device-based pelvic tracking may provide opportunities for future investigations of intraoperative pelvic motion during THA. Although continuous intraoperative pelvic orientation data were not stored in the present study, the ability to continuously monitor pelvic orientation using a wearable platform may facilitate future research in this area.

This study had some limitations. First, this was a single-center retrospective study with a relatively small sample size of 30 hips from 27 patients. Second, no direct comparison with existing navigation systems was performed because the primary objective of this study was to evaluate the feasibility of the smartwatch-based pelvic tracking concept, rather than comparative accuracy. Third, the postoperative cup orientation measurements were performed by a single observer, although excellent intra-observer reproducibility was demonstrated. Fourth, the additional technical validation was performed under controlled bench conditions and may not fully reproduce intraoperative conditions. Fifth, continuous intraoperative pelvic orientation data were not stored, precluding quantitative evaluation of intraoperative pelvic motion. Finally, the short follow-up period precludes conclusions regarding clinical safety and longer-term outcomes. Further studies with larger patient cohorts and comparative designs are warranted.

## Conclusions

We successfully implemented a smartwatch-based pelvic tracking system integrated with AR navigation for THA. The system achieved a preliminary range of cup-positioning accuracy without requiring continuous optical visualization of a pelvic reference marker while utilizing a compact, commercially available wearable device. These findings support the concept that consumer wearable technology can be successfully integrated into pelvic tracking systems and may represent a promising direction for future navigation in arthroplasty.

## Supplementary Information

Supplementary Material 1.

## Data Availability

The datasets generated and/or analyzed during the current study are available from the corresponding author on reasonable request.

## Abbreviations

AR: Augmented reality
ASIS: Anterior superior iliac spine
CI: Confidence interval
FPP: Functional pelvic plane
RA: Radiographic anteversion
RI: Radiographic inclination
THA: Total hip arthroplasty
