# Supplementary material for: Smartwatch-based pelvic tracking system for augmented reality navigation in total hip arthroplasty: a preliminary clinical study
Source: Arthroplasty. 2026 Nov 2;8:82. doi: 10.1186/s42836-026-00441-3 (PMC13726019; doi:10.1186/s42836-026-00441-3)
Supplement: Supplementary file 1 [file 42836_2026_441_MOESM1_ESM.docx]

**Supplementary Material**

**Smartwatch-based pelvic tracking system for augmented reality navigation in total hip arthroplasty: a preliminary clinical study**

Ryohei Takada, Ryo Inokuchi, Yusuke Amano, Riko Yamashita, Toshitaka Yoshii, Hideyuki Koga

**Supplementary Methods**

**Detach-reattach reproducibility test**

To evaluate the reproducibility of the dedicated smartwatch fixation mechanism, a bench test was performed using a pelvic model with the smartwatch fixation device rigidly secured to the iliac crest (Supplementary Fig. S1). The pelvic model and the other components of the experimental setup were maintained in fixed positions throughout the experiment. After establishing the initial reference orientation, the smartwatch was detached from and reattached to the fixation device 20 consecutive times. Following each reattachment, the three-dimensional orientation of the smartwatch and the navigation-derived RI and RA values were recorded. Differences in smartwatch orientation and RI and RA were calculated relative to the initial reference measurements. The mean ± standard deviation and maximum absolute difference were calculated for each measurement.

**Time-dependent drift and FPP invocation test**

To evaluate time-dependent relative orientation drift between the smartwatch and smartphone and the effect of FPP invocation, a bench test was performed under static conditions. The pelvic model, smartwatch, smartphone, and cup impactor were maintained in fixed positions throughout each trial. After initial FPP registration, the orientation at the start of each trial was defined as the reference orientation. Measurements were obtained at 10, 30, and 60 min after establishment of the initial reference, with three independent trials performed at each time point. For each independent trial, FPP registration and establishment of the initial reference were repeated from the beginning. At each time point, the navigation-derived RI and RA values were recorded immediately before FPP invocation. FPP invocation was then performed, and RI and RA were recorded again immediately after invocation. Differences from the initial reference values were calculated before and after FPP invocation to evaluate time-dependent relative orientation drift and the extent to which FPP invocation restored the initial orientation relationship.

**Supplementary Table S1.** Reproducibility of smartwatch orientation and navigation-derived cup orientation after repeated detachment and reattachment

| **Parameter** | **Mean difference ± SD (°)** | **Maximum absolute difference (°)** |
| --- | --- | --- |
| α | +1.25 ± 0.64 | 2.0 |
| β | −0.10 ± 0.31 | 1.0 |
| γ | 0.00 ± 0.32 | 1.0 |
| RI | −0.86 ± 0.40 | 1.4 |
| RA | +0.10 ± 0.28 | 0.9 |

**Footnote:** Differences were calculated relative to the initial reference measurement after each of 20 consecutive detach–reattach cycles. Positive and negative values indicate the direction of change relative to the initial reference. RI, radiographic inclination; RA, radiographic anteversion; SD, standard deviation.

**Supplementary Table S2.** Time-dependent differences in navigation-derived cup orientation before and after FPP invocation

| **Time point** | **RI before invocation (°)** | **RI after invocation (°)** | **RA before invocation (°)** | **RA after invocation (°)** |
| --- | --- | --- | --- | --- |
| 10 min | 1.23 ± 0.71 | −0.40 ± 0.70 | −0.03 ± 0.06 | 0.10 ± 0.10 |
| 30 min | 2.03 ± 1.00 | 0.80 ± 0.60 | 0.00 ± 0.53 | 0.27 ± 0.64 |
| 60 min | 2.27 ± 0.96 | 0.63 ± 0.86 | 0.77 ± 0.78 | −0.13 ± 0.65 |

**Footnote:** Values are presented as mean difference ± standard deviation from the initial reference measurement. Three independent trials were performed at each time point. For each independent trial, FPP registration and establishment of the initial reference were repeated from the beginning. Measurements were obtained immediately before and after FPP invocation. Positive and negative values indicate the direction of change relative to the initial reference. FPP, functional pelvic plane; RI, radiographic inclination; RA, radiographic anteversion.

**Fig. S1.** Smartwatch fixation mechanism used for the detach–reattach reproducibility test. (A) Smartwatch attached to the dedicated fixation device secured to the iliac crest of the pelvic model. (B) Smartwatch detached from the fixation device while the pelvic-side fixation components remained in place.

**Fig. S2.** Experimental setup and navigation display used for the technical validation. (A) Overall experimental setup showing the pelvic model with the smartwatch fixation device, smartphone, and cup impactor with the attached AR marker. (B) Enlarged view of the smartphone display during the bench test.
